# Prevalence of fear of falling and associated factors among Japanese community-dwelling older adults

**DOI:** 10.1097/MD.0000000000009721

**Published:** 2018-01-26

**Authors:** Yoshihito Tomita, Kazuhiko Arima, Ritsu Tsujimoto, Shin-ya Kawashiri, Takayuki Nishimura, Satoshi Mizukami, Takuhiro Okabe, Natsumi Tanaka, Yuzo Honda, Kazumi Izutsu, Naoko Yamamoto, Izumi Ohmachi, Mitsuo Kanagae, Yasuyo Abe, Kiyoshi Aoyagi

**Affiliations:** a Department of Public Health; b Department of Rehabilitation, Nishi-Isahaya Hospital, Isahaya; c Department of Orthopedic Surgery; d Department of Community Medicine; e Department of Health Science, Faculty of Medicine Kagoshima University, Kagoshima; f Department of Health Science, Nagasaki University Graduate School of Biomedical Sciences, Nagasaki, Japan.

**Keywords:** falls in the previous year, pain management, physical function

## Abstract

To determine the prevalence of fear of falling and associated factors among Japanese community-dwelling older adults.

Cross-sectional study between 2011 and 2013.

Community in which residents voluntarily attended a health examination.

We recruited 844 older adults (male, n = 350; female, n = 494) aged 60 to 92 years from among those who presented at the health examination.

We assessed fear of falling, falls in the previous year, pain, comorbidity, and cataracts. Five times chair stand time was applied as an indicator of physical performance.

The prevalence of fear of falling was 26.9% and 43.3% among the men and women, respectively. Men and women who feared falling were older (*P* < .01), had longer 5 times chair stand time (*P* < .01), and more falls in the previous year (*P* < .05), pain (*P* < .01), and comorbidity (*P* < .05). Multivariate logistic regression analysis identified advanced age (odds ratios [OR], 1.57; 95% confidence interval [CI], 1.03–2.39), falls in the previous year (OR, 2.44; 95%CI, 1.29–4.64), and pain (OR, 1.82; 95%CI, 1.03–3.22) in men, and advanced age (OR, 1.59; 95%CI, 1.13–2.24), longer 5 times chair stand times (OR, 1.28; 95%CI, 1.04–1.59), falls in the previous year (OR, 2.59; 95%CI, 1.54–4.34), and pain (OR, 1.65; 95%CI, 1.06–2.55) in women as being independently associated with fear of falling.

The prevalence of fear of falling was similar to previous reports. Advanced age, falls in previous year, and pain were associated with fear of falling in men. A longer 5 times chair stand time was also associated with fear of falling among older adult women. Maintenance of physical function and pain management might be important for older adults with fear of falling.

## 1 Introduction

Fear of falling is a fundamental health problem among community-dwelling older adults.^[1]^ Such fear is reportedly associated with other health issues, such as reduced levels of physical activity,^[2]^ reduced ability to perform activities of daily living (ADL),^[3,4]^ increased risk of admission to a care institution for older adults within 12 months,^[4]^ and decreased quality of life (QOL).^[5]^ Fear of falling commonly arises after a fall,^[6]^ but it can also occur without a history of falls.^[7]^ Fear of falling is not only the immediate result of falls but also a risk factor for falls,^[8]^ which creates a vicious circle between falls and fear of falling. Identifying related factors to identify candidates for intervention is an indispensable step towards attenuating fear of falling.

The reported prevalences of fear of falling among community dwellers are 29% in the USA,^[9]^ 57.9% in Japan,^[10]^ and 76.6% in Korea.^[11]^ The inconsistencies among the reported prevalences of fear of falling might be associated with differences in population characteristics, such as age distribution, fall history, frailty, or culture.^[11–13]^ Chang et al^[14]^ reported a higher rate of fear of falling among women and a sex influence on fear of falling. Therefore, we separately analyzed fear of falling between Japanese community-dwelling older men and women.

Previous studies have shown that factors such as age, sex, physical performance, comorbidity, a history of falls, and visual impairment affect fear of falling.^[15–17]^ Physical performance is associated with fear of falling.^[12,15,18]^ Poor performance in 5 times chair stand is associated with fear of falling, suggesting that overall lower limb strength and deficits in dynamic postural control are contributing factors.^[18]^ Some investigators have identified a relationship between fear of falling and pain,^[19,20]^ whereas others have not.^[5,21]^ Fear of falling is associated with comorbidities^[22]^ such as cardiovascular diseases, diabetes mellitus, stroke,^[14]^ and chronic obstructive pulmonary disease.^[23]^ Fear of falling in older adults with cataracts is more prevalent when the visual disability is worse.^[24]^ Few studies have investigated the factors associated with fear of falling among Japanese community-dwelling older adults.

It would be useful to know which factors are associated with the fear of falling for development of health strategies to reduce the fear of falling. The present study aimed to determine the prevalence of and factors associated with fear of falling, namely age, performance in the 5 times chair stand time, falls in the previous year, pain, comorbidities, and cataracts among Japanese community-dwelling older men and women.

## 2 Participants and methods

Community-dwelling older adults aged 60 to 92 years in Nagasaki Prefecture, Japan were invited to participate in health examinations between August 2011 and November 2013. We recruited 844 of them (350 men and 494 women) who were non-institutionalized, lived independently, and had sufficient cognitive function to respond to a questionnaire. We excluded persons who were unable to stand up from chair alone.

The participants were asked the question, “Are you afraid of falling?” to determine the prevalence of fear of falling. Self-administered questionnaires included information on falls in the previous year, lumbar pain, knee pain, comorbidities, and cataracts. The questionnaires were handed in advance before health examination. We collected them after the researcher checked the answers. Lumbar pain and/or knee pain on most days during the previous month was categorized as pain. Participants were asked if they had any comorbidities (heart disease, lung disease, stroke, or diabetes mellitus).

Height (m) and weight (kg) were measured in participants wearing light clothing and without shoes. Body mass index (BMI) was calculated as weight divided by height squared (kg/m^2^).

We measured the amount of time required to compete the 5 times chair stand time, in which the participants first placed their arms across their chest and stood upright once from a sitting position. If successful, they sequentially stood upright and sat down as quickly as possible 5 times, and time was stopped at the final upright position at the end of the fifth repeat. This procedure was completed twice and the 2 times were averaged.

The Nagasaki University Ethics Committee approved the study, for which all recruits provided written informed consent to participate before undergoing the health examination.

### 2.1 Statistical analysis

Comparisons between men and women and among men and women with and without fear of falling proceeded using Student's *t* test for continuous variables or the chi-square test for nominal variables. Trends in the prevalence of fear of falling among age groups (60–69, 70–79, and ≥80 years) were evaluated using the Mantel–Haenszel test.

Associations between fear of falling and age, 5 times chair stand time, falls in the previous year, and pain were evaluated using logistic regression models to estimate odds ratios (OR) and 95% confidence intervals (CI) in men and women, respectively. Variables were selected according to the Akaike information criterion. Differences between observed and predicted prevalences in multivariate logistic regression analyses were evaluated using Hosmer–Lemeshow tests. A probability of *P* < .05 was considered to indicate significance. All data were statistically analyzed using SPSS software version 20 (SPSS Inc., Chicago, IL).

## 3 Results

Table 1 shows the characteristics of the male and female participants. The mean ages of the men and women were 70.1 ± 6.4 and 69.8 ± 6.1 years, respectively. The mean BMI was greater in men than in women (*P* < .001). Among the men and women, 14% and 18% respectively reported having experienced at least 1 fall during the previous year. Pain and cataracts were more prevalent among the women (*P* = .032 and *P* = .03, respectively). The number of participants who have at least 1 comorbidity was more prevalent among the men (*P* = .001).

**Table 1:**
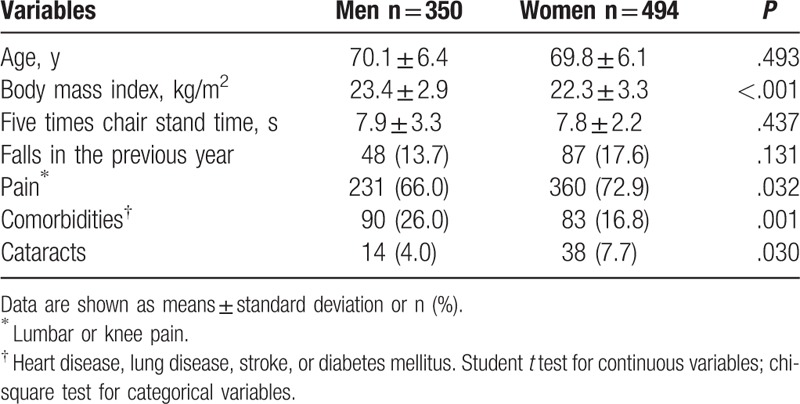
Characteristics of participants (n = 844).

Table 2 shows the prevalence of fear of falling according to age in men and women. The overall prevalence was greater among women than men (43.3% vs 26.9%, *P* < .001) and increased with age in both sexes (both *P* < .001).

**Table 2:**
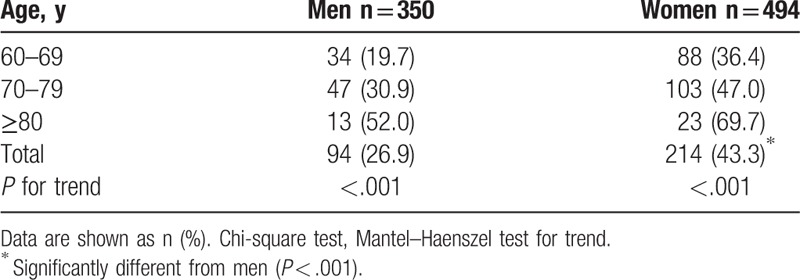
Prevalence of fear of falling by age in men and women (n = 844).

Table 3 separately compares age, BMI, 5 times chair stand time, falls in the previous year, pain, comorbidity, and cataracts in men and in women with and without fear of falling. Both men and women with fear of falling had longer 5 times chair stand times and higher prevalence of falls in the previous year, pain, and comorbidity (all *P* < .05), whereas the prevalences of BMI and cataracts did not significantly differ.

**Table 3:**
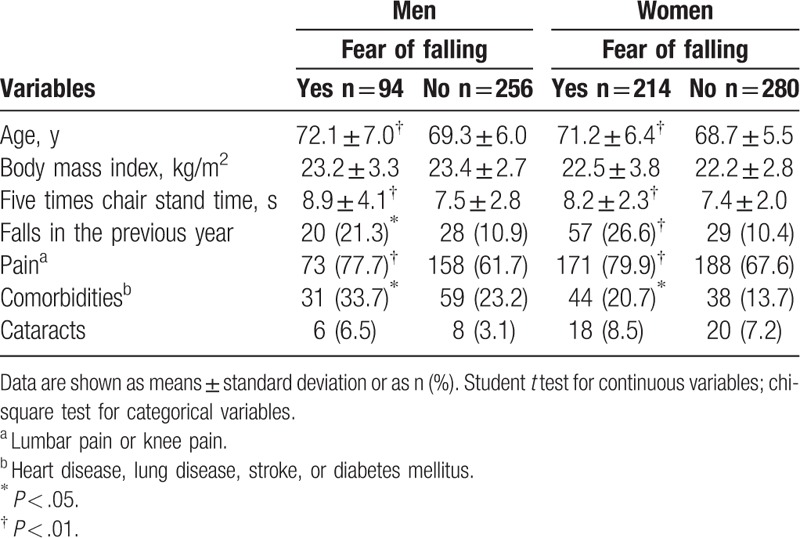
Comparison of age, body mass index, 5 times chair stand time, falls in the previous year, pain, comorbidity, and cataracts between men and women with and without fear of falling (n = 844).

Table 4 shows the results of multivariate logistic regression to assess factors associated with fear of falling in men and women. The Hosmer–Lemeshow test found no significant difference between the observed and predicted prevalences. More advanced age, falls in the previous year, and pain were independently and significantly associated with fear of falling in both men and in women. Longer 5 times chair stand times were also significantly associated with fear of falling in women.

**Table 4:**
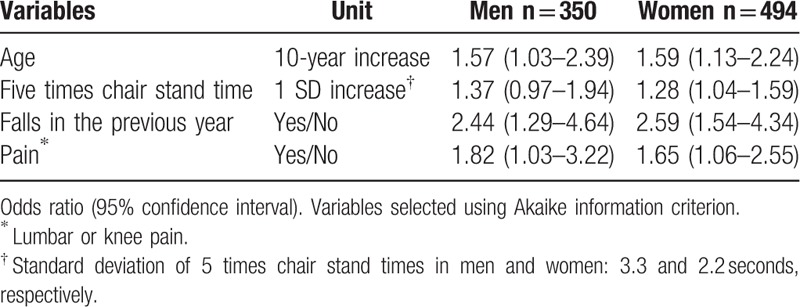
Independent associations with fear of falling in multivariate model (n = 844).

## 4 Discussion

The present study showed that the prevalence of fear of falling increased with age in both sexes, which was similar to previous findings,^[25–27]^ and that it was significantly higher in women than in men. Several studies have shown a higher prevalence of fear of falling among older adult women than men.^[9,28–32]^ This could be because women tend to develop osteoporosis and a weaker musculoskeletal system more frequently than men.^[5]^

Fear of falling often develops after experiencing a fall.^[6]^ We found that falls in the previous year were significantly associated with fear of falling in both sexes. Having had at least 1 fall is an independent risk factor for developing fear of falling after adjustment for age, sex, and vision problems.^[21,31]^ Developing a fear of falling can cause older adults to avoid physical activity, experience more difficulty with activities of daily living, and become less able to perform exercises to improve muscle strength and postural control.^[13]^ This pattern might fuel further fear and avoidance, and cause further deterioration of physical performance and increased risk of falls in the long term.^[13]^ Interventions for preventing falls in older people living in the community would be important.^[32]^

We found that fear of falling was associated with longer 5 times chair stand times in women, indicating less muscle strength in the lower extremities, but not in men. Previous studies have shown a relationship between fear of falling and a need for more time to complete the 5 times chair stand test.^[12,18,33]^ These findings indicated that older adults should maintain muscle strength in the lower extremities to reduce the fear of falling. The association in men might not have reached significance in the preset study due to a relatively smaller sample size (OR was greater for men than for women: 1.37 vs 1.28). Since older adult men and women with functional dependence both tend to have fear of falling, assessment and intervention are necessary for both sexes.^[5]^ Maintenance of physical function among older adults might be improved to reduce fear of falling.

Several reports have described a relationship between fear of falling and pain.^[19,34]^ We also found that pain was significantly associated with fear of falling in both sexes. Pain might increase the risk of developing fear of falling among older adults.^[20]^ Pain, particularly in the lower back, hips, knees, and feet, can alter gait biomechanics and postural stability,^[35]^ which over time might increase feelings of unsteadiness and fear of falling.^[36]^ Clinicians working with older adults who have pain should consider assessing fear of falling and, if necessary, intervene when an individual at risk is identified.^[20]^ Pain management might be important for older adults with fear of falling.

Univariate analysis in the present study associated comorbidity with fear of falling, but the multivariate model did not. Chronic morbidity is associated with fear of falling.^[17,22]^ We did not identify an association between fear of falling and cataracts, although visual impairment has been associated.^[16,17,25]^ Because we used cataracts as a surrogate for visual impairment, we might have missed some visual malfunctions. We also might have underestimated associations with fear of falling. Associations between fear of falling and comorbidity or visual impairment should be explored in a future study.

Study limitations include the following. The results of this cross-sectional analysis did not necessarily identify causal relationships. Longitudinal studies are required to establish causal relationships between fear of falling and physical performance or pain. The study participants were recruited from among community-dwelling residents who voluntarily attended a health examination. Participants with poorer health were not included, which might have affected the results. Because they may be difficult to answer the severity, the severity of comorbidities was not estimated. Data about depression or cognitive function were not available. Thus, these limitations might have contributed to an underestimation of associations. Our findings were generated from Japanese participants. Therefore, the results cannot be extrapolated to other ethnicities.

We showed that more advanced age, falls in the previous year, and pain were independently associated with fear of falling in Japanese community-dwelling older adults. A longer 5 times chair stand time was also associated with fear of falling among older adult women. Maintenance of physical function and pain management might be important for older adults with fear of falling. However, our study has not demonstrated whether such an intervention is effective in reducing the fear of falling or not. Further study is needed to assess the effectiveness.

## 5 Author contributions

Study design (YT, KA, YA, KA), data collection (YT, KA, RT, SK, TN, SM, TO, KI, NY, IO, MK, YA, KA), data analysis (YT, KA, TN, NT, YH, MK, YA, KA), writing of the manuscript (YT, KA), editing the manuscript (MK, KA), study supervision (KA).

## References

[1] Jorstad EC Hauer K Becker C Measuring the psychological outcomes of falling: a systematic review. J Am Geriatr Soc 2005;53:501–10. 15743297 10.1111/j.1532-5415.2005.53172.x

[2] Bruce DG Devine A Prince RL Recreational physical activity levels in healthy older women: the importance of fear of falling. J Am Geriatr Soc 2002;50:84–9. 12028251 10.1046/j.1532-5415.2002.50012.x

[3] Tinetti ME Mendes de Leon CF Doucette JT Fear of falling and fall-related efficacy in relationship to functioning among community-living elders. J Gerontol 1994;49:M140–7. 8169336 10.1093/geronj/49.3.m140

[4] Cumming RG Salkeld G Thomas M Prospective study of the impact of fear of falling on activities of daily living, SF-36 scores, and nursing home admission. J Gerontol A Biol Sci Med Sci 2000;55:M299–305. 10819321 10.1093/gerona/55.5.m299

[5] Suzuki M Ohyama N Yamada K The relationship between fear of falling, activities of daily living and quality of life among elderly individuals. Nurs Health Sci 2002;4:155–61. 12406202 10.1046/j.1442-2018.2002.00123.x

[6] Aoyagi K Ross PD Davis JW Falls among community-dwelling elderly in Japan. J Bone Miner Res 1998;13:1468–74. 9738520 10.1359/jbmr.1998.13.9.1468

[7] Luukinen H Koski K Kivela SL Social status, life changes, housing conditions, health, functional abilities and life-style as risk factors for recurrent falls among the home-dwelling elderly. Public Health 1996;110:115–8. 8901255 10.1016/s0033-3506(96)80057-6

[8] Friedman SM Munoz B West SK Falls and fear of falling: which comes first? A longitudinal prediction model suggests strategies for primary and secondary prevention. J Am Geriatr Soc 2002;50:1329–35. 12164987 10.1046/j.1532-5415.2002.50352.x

[9] Arfken CL Lach HW Birge SJ The prevalence and correlates of fear of falling in elderly persons living in the community. Am J Public Health 1994;84:565–70. 8154557 10.2105/ajph.84.4.565 PMC1614787

[10] Sakurai R Fujiwara Y Yasunaga M [Association of confidence in motor function and fear of falling with physical ability in community-dwelling older people]. Nihon Ronen Igakkai Zasshi 2013;50:369–76. 23979344 10.3143/geriatrics.50.369

[11] Kim S So WY Prevalence and correlates of fear of falling in Korean community-dwelling elderly subjects. Exp Gerontol 2013;48:1323–8. 24001938 10.1016/j.exger.2013.08.015

[12] Tomita Y Arima K Kanagae M Association of physical performance and pain with fear of falling among community-dwelling Japanese women aged 65 years and older. Medicine (Baltimore) 2015;94:e1449. 26334906 10.1097/MD.0000000000001449 PMC4616514

[13] Delbaere K Crombez G Vanderstraeten G Fear-related avoidance of activities, falls and physical frailty. A prospective community-based cohort study. Age Ageing 2004;33:368–73. 15047574 10.1093/ageing/afh106

[14] Chang HT Chen HC Chou P Factors associated with fear of falling among community-dwelling older adults in the Shih-Pai Study in Taiwan. PLoS One 2016;11:e0150612. 26933882 10.1371/journal.pone.0150612 PMC4775068

[15] Murphy SL Williams CS Gill TM Characteristics associated with fear of falling and activity restriction in community-living older persons. J Am Geriatr Soc 2002;50:516–20. 11943049 10.1046/j.1532-5415.2002.50119.x PMC3046411

[16] Scheffer AC Schuurmans MJ van Dijk N Fear of falling: measurement strategy, prevalence, risk factors and consequences among older persons. Age Ageing 2008;37:19–24. 18194967 10.1093/ageing/afm169

[17] Kempen GI van Haastregt JC McKee KJ Socio-demographic, health-related and psychosocial correlates of fear of falling and avoidance of activity in community-living older persons who avoid activity due to fear of falling. BMC Public Health 2009;9:170. 19490640 10.1186/1471-2458-9-170 PMC2698855

[18] Deshpande N Metter EJ Bandinelli S Psychological, physical, and sensory correlates of fear of falling and consequent activity restriction in the elderly: the InCHIANTI study. Am J Phys Med Rehabil 2008;87:354–62. 18174852 10.1097/PHM.0b013e31815e6e9b PMC2495025

[19] Hubscher M Vogt L Schmidt K Perceived pain, fear of falling and physical function in women with osteoporosis. Gait Posture 2010;32:383–5. 20663672 10.1016/j.gaitpost.2010.06.018

[20] Stubbs B West E Patchay S Is there a relationship between pain and psychological concerns related to falling in community dwelling older adults? A systematic review. Disabil Rehabil 2014;36:1931–42. 24467675 10.3109/09638288.2014.882419

[21] Howland J Lachman ME Peterson EW Covariates of fear of falling and associated activity curtailment. Gerontologist 1998;38:549–55. 9803643 10.1093/geront/38.5.549

[22] Dierking L Markides K Al Snih S Fear of falling in older Mexican Americans: a longitudinal study of incidence and predictive factors. J Am Geriatr Soc 2016;64:2560–5. 27783403 10.1111/jgs.14496 PMC5173438

[23] Beauchamp MK Sibley KM Lakhani B Impairments in systems underlying control of balance in COPD. Chest 2012;141:1496–503. 22116798 10.1378/chest.11-1708

[24] Palagyi A Ng JQ Rogers K Fear of falling and physical function in older adults with cataract: exploring the role of vision as a moderator. Geriatr Gerontol Int 2017;17:1551–8. 27917612 10.1111/ggi.12930

[25] Bertera EM Bertera RL Fear of falling and activity avoidance in a national sample of older adults in the United States. Health Soc work 2008;33:54–62. 18326450 10.1093/hsw/33.1.54

[26] Evitt CP Quigley PA Fear of falling in older adults: a guide to its prevalence, risk factors, and consequences. Rehabil Nurs 2004;29:207–10. 15597999

[27] Zijlstra GA van Haastregt JC van Eijk JT Prevalence and correlates of fear of falling, and associated avoidance of activity in the general population of community-living older people. Age Ageing 2007;36:304–9. 17379605 10.1093/ageing/afm021

[28] Oya T Uchiyama Y Shimada H [Factors associated with fear of falling among community-dwelling elderly adults without reduced performance in instrumental activities of daily living]. Nihon Ronen Igakkai Zasshi 2012;49:457–62. 23269025 10.3143/geriatrics.49.457

[29] Uemura K Shimada H Makizako H A lower prevalence of self-reported fear of falling is associated with memory decline among older adults. Gerontology 2012;58:413–8. 22488205 10.1159/000336988

[30] Lachman ME Howland J Tennstedt S Fear of falling and activity restriction: the survey of activities and fear of falling in the elderly (SAFE). J Gerontol B Psychol Sci Soc Sci 1998;53:P43–50. 9469171 10.1093/geronb/53b.1.p43

[31] Oh-Park M Xue X Holtzer R Transient versus persistent fear of falling in community-dwelling older adults: incidence and risk factors. J Am Geriatr Soc 2011;59:1225–31. 21718266 10.1111/j.1532-5415.2011.03475.x PMC3298667

[32] Gillespie LD Robertson MC Gillespie WJ Interventions for preventing falls in older people living in the community. Cochrane Database Syst Rev 2012;12:CD007146. 10.1002/14651858.CD007146.pub3 PMC8095069 22972103

[33] Rochat S Bula CJ Martin E What is the relationship between fear of falling and gait in well-functioning older persons aged 65 to 70 years? Arch Phys Med Rehabil 2010;91:879–84. 20510978 10.1016/j.apmr.2010.03.005

[34] Martin FC Hart D Spector T Fear of falling limiting activity in young-old women is associated with reduced functional mobility rather than psychological factors. Age Ageing 2005;34:281–7. 15863412 10.1093/ageing/afi074

[35] Lihavainen K Sipila S Rantanen T Contribution of musculoskeletal pain to postural balance in community-dwelling people aged 75 years and older. J Gerontol A Biol Sci Med Sci 2010;65:990–6. 20403947 10.1093/gerona/glq052

[36] Patel KV Phelan EA Leveille SG High prevalence of falls, fear of falling, and impaired balance in older adults with pain in the United States: findings from the 2011 National Health and Aging Trends Study. J Am Geriatr Soc 2014;62:1844–52. 25283473 10.1111/jgs.13072 PMC4206582

